# Systematic identification of phosphorylation-mediated protein interaction switches

**DOI:** 10.1371/journal.pcbi.1005462

**Published:** 2017-03-27

**Authors:** Matthew J. Betts, Oliver Wichmann, Mathias Utz, Timon Andre, Evangelia Petsalaki, Pablo Minguez, Luca Parca, Frederick P. Roth, Anne-Claude Gavin, Peer Bork, Robert B. Russell

**Affiliations:** 1 CellNetworks, Bioquant, University of Heidelberg, Im Neuenheimer Feld 267, Heidelberg, Germany; 2 Biochemie Zentrum Heidelberg (BZH), Im Neuenheimer Feld 328, Heidelberg, Germany; 3 Lunenfeld-Tanenbaum Research Institute, Mount Sinai Hospital, 600 University Avenue, Toronto, Ontario, Canada; 4 European Molecular Biology Laboratory, Meyerhofstrasse 1, Heidelberg, Germany; 5 Donnelly Centre and Departments of Molecular Genetics and Computer Science, University of Toronto, Toronto, Ontario, Canada; 6 Center for Cancer Systems Biology, Dana-Farber Cancer Institute, One Jimmy Fund Way, Boston, Massachusetts, United States; 7 Canadian Institute for Advanced Research, Toronto, Ontario, Canada; University of California San Diego, UNITED STATES

## Abstract

Proteomics techniques can identify thousands of phosphorylation sites in a single experiment, the majority of which are new and lack precise information about function or molecular mechanism. Here we present a fast method to predict potential phosphorylation switches by mapping phosphorylation sites to protein-protein interactions of known structure and analysing the properties of the protein interface. We predict 1024 sites that could potentially enable or disable particular interactions. We tested a selection of these switches and showed that phosphomimetic mutations indeed affect interactions. We estimate that there are likely thousands of phosphorylation mediated switches yet to be uncovered, even among existing phosphorylation datasets. The results suggest that phosphorylation sites on globular, as distinct from disordered, parts of the proteome frequently function as switches, which might be one of the ancient roles for kinase phosphorylation.

## Author summary

Most biological processes occur by molecules connecting to other molecules, and the precise details of these connections can often be seen in their three-dimensional structures or inferred from those of similar molecules. The ways in which molecules fit together are often affected and regulated by small chemical modifications to the structures of the molecules. Thousands of these modifications have been found in large-scale experiments, without knowing what connections they might affect or how. Some make molecules fit together better and some make the fit worse. We have combined 3D structures with data for a particular type of modification known as 'phosphorylation' to predict these effects and have found more than a thousand phosphorylations that may strengthen or weaken molecular connections, thereby allowing us to explain how certain biological processes are regulated.

## Introduction

Protein phosphorylation is important for many cellular processes, including signalling (e.g. [1]), transcription (e.g. [2]) and metabolism (e.g. [3]). Many phosphorylation sites act as switches to regulate inter-protein interactions (e.g. [4]) and there have been many studies into mechanisms, specificities and structures of kinases, phosphatases (e.g. [5,6]) and recognition domains (SH2, 14-3-3, etc.) that regulate or bind them (e.g. [7,8]). Phosphosites also regulate enzymatic function (e.g. [9]), target proteins for degradation (e.g. [10]) and play many other intriguing roles, e.g. in ultrasensitivity of Sic1/Cdc4 interactions [11] or in RNA polymerase II recognition during mRNA processing [12].

High-throughput efforts have identified thousands of phosphosites in many biological systems [13–16]. Few of them overlap with those identified in low-throughput studies (e.g. [17]) meaning that the molecular consequences of phosphorylation are not understood for most sites. Previous analyses have shown functional sites to be generally conserved [18] and over-represented in disordered regions [19,20]. Functional phosphosites have been proposed to have evolved from negatively charged amino acids, by making charge-mediated protein interactions tunable by kinases [21]. Functional coupling and/or co-evolution of sites has been suggested to be an important determinant of protein function [20,22], with codes of post-translational modifications refining protein function, for example in transcription factors [23,24]. While many important proteins are known to be modified at multiple sites, the functional implications of these codes are understood for only a handful.

There are now many thousands of three-dimensional (3D) structures of protein-interactions [25–28], providing an invaluable resource to study molecular mechanisms. These include structures of phosphorylated proteins and structures on which phosphosites from homologous proteins can be modelled. Phosphosites in known structures tend to be conserved when they occur at interfaces and only a minority of these alter binding affinity[29]. Mechanistic investigations show that certain phosphosites target interfaces, thus enabling predictions of function (e.g. [30]). The now increased volume of both phosphoproteomic and 3D structure data provides an opportunity to study and predict the mechanistic impact of phosphosites on protein interfaces. Accordingly, we present here an approach to identify potential phosphosite switches, using structures of phosphorylated proteins and of their homologues, and to predict whether they turn interactions on or off. From a large phosphosite dataset we predict hundreds of new switches, a selection of which, via mutations to phosphomimics, we demonstrate are likely responsible for mediating protein-protein interactions.

## Results

### A dataset of phosphosites

**Fig 1:**
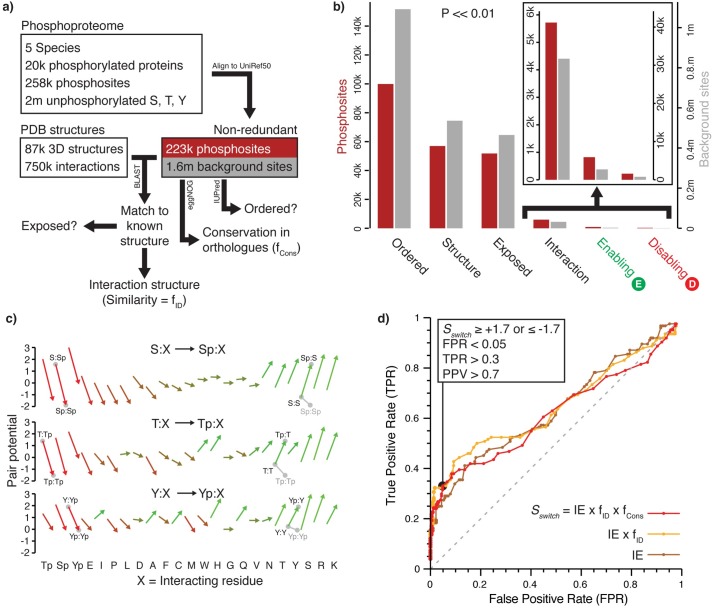
Summary of the data processing pipeline and results. ***a)*** Source data and processing steps. ***b)*** Summary of counts of phosphosites (red bars, left-hand y-axis) and background sites (grey bars, right-hand y-axis) that are ordered, matched to a template structure, were surface exposed, in an interaction interface, and predicted to be enabling or disabling by *S*_*switch*_. The left and right-hand axes are scaled to the total number of non-redundant phosphosites and background sites, respectively. The difference between the fractions of phosphosites and background sites for all categories is significant (P << 0.01). ***c)*** Change in residue interaction pair-potentials of Serine, Threonine and Tyrosine upon phosphorylation. Grey lines emphasise change in residue interaction potentials of a residue pointing at a second copy of itself across a homodimeric interface when both copies are phosphorylated. ***d)*** Receiver Operator Characteristic (ROC) curves showing the ability of *S*_*switch*_ to identify enabling or disabling effects of known phosphosite switches, and the change in performance by including the interaction effect (IE, = sum of pair-potentials), similarity of the query to the structure template (f_ID_), and the conservation of the site across orthologues (f_Cons_). Note that the Enabling and Disabling labels in (b) are defined using the thresholds in (d).

To search for new potential switches we used a processed dataset of 223,971 phosphosites in 19,483 proteins from five organisms, defining the 1.6 million to date unphosphorylated Serine, Threonine and Tyrosine residues in the same proteins as background (Fig 1A). The vast majority of known sites (>90%) come only from high-throughput studies, meaning their particular functions and consequences have not been studied in any detail. The majority (55%) of the phosphosites are in disordered regions, as noted previously [19,31], which is significantly higher than the background (Fig 1B, 32%, P << 0.01). 56,209 sites (25%), including 8341 (7%) of those in disordered regions, could be matched to 3D structures, either of the protein itself or a homolog [32]. 8714 (16%) phosphosites were within contacting distance of a small molecule (more than background: 16% vs 13% P << 0.01), including some known enzymatic switches (e.g. [33]), though the majority have no known functional role. Whether these sites are regulatory or trapped phosphoenzyme intermediates requires additional investigation.

Phosphosites are more likely to lie on protein surfaces (90% vs 87%, P << 0.01, Figs 1B & S1), to be at protein-protein interaction interfaces (10% vs 6%, P << 0.01) and, when at an interface, to be conserved or aligned to Aspartate or Glutamate in orthologues (P << 0.01, S2 Fig). A total of 34 sites at interfaces are aligned to at least 50% Aspartate/Glutamate residues, supporting the idea (e.g. [21]) that some sites have evolved from negative residues to modulate protein interactions. Only 1455 sites (0.7%) are matched to phosphorylated residues visible in at least one 3D structure and only 122 of these are at interaction interfaces (i.e. potential switches), emphasizing that few sites are understood in any mechanistic detail.

### Defining and predicting enabling and disabling phosphosite switches

We defined phosphosite-switches as Serine, Threonine and Tyrosine residues in protein interfaces that make interactions stronger (enabling) or weaker (disabling) through interplay between the physicochemical properties of the modification and the interface. To identify such sites we first computed a set of pair-potential scores that compare the frequency of pairs of contacting residues at interfaces to a random model (Fig 1C, S5 Table), summed the differences in scores between phosphorylated and unmodified residues to give the Interaction Effect (IE), and defined enabling as those where the IE increases upon phosphorylation (i.e. a better interaction according to statistical preferences) and disabling where it decreases [32].

Accuracy of interface structures is proportional to the sequence similarity between the protein of interest and the 3D template used to model it [28], and our identified sites span the entire range of sequence identities. Similarly, the likelihood that a phosphosite is a true switch will increase with the degree to which it is conserved across orthologous sequences [20]. To account for both of these effects, we multiplied IE by the similarity between the protein and the 3D template (fraction of identical residues, f_ID_) and the site conservation across orthologues (fraction of residues that are either conserved or Aspartate or Glutamate, f_Cons_) to give an overall score *S*_*switch*_, where high positive/negative values indicate the best switch candidates.

We benchmarked *S*_*switch*_ using known phosphosite-switched interactions extracted from UniProt and PhosphoSitePlus [34]. These sets are biased towards enabling sites (S1 Table) since most sites are related to gain of interaction upon phosphorylation. Incorporation of the measures of structural match quality and residue conservation improves performance, though only marginally, perhaps reflecting the variability of sites and the relatively weak conservation of sites outside of closely related species (Fig 1D). We also observed that absolute *S*_*switch*_ is better able to find any effect, disabling or enabling, than are structural match quality and residue conservation by themselves (S3 Fig), suggesting that conserved phosphosites seen directly in protein-protein interfaces may play roles other than switching. Values of *S*_*switch*_ ≥ 1.7 or ≤ -1.7 give a false positive rate = 0.05 with reasonable sensitivity (= 0.35), positive predictive value (> 0.78) and accuracy (0.74), and a very low p-value (<< 1 x 10^−6^) (Fig 1D, S4 Fig, S6 Table).

Attempts to improve performance using logistic regression (see Methods) slightly reduced the sensitivity to 0.33 (but with the same accuracy) at our desired false positive rate (0.05; See S4 Fig and S6 Table). We believe this to be a function of the small benchmark rather than any issue with the regression approach; a larger benchmark would likely lead to an improved performance.

To check for possible bias towards enabling sites from kinase-substrate interactions, we removed kinase interactors from the benchmark set (see Methods) and re-calculated the benchmark statistics, resulting in a slightly increased sensitivity (0.39) and the same accuracy (for the desired false positive rate (0.05; See S7 Table) at the cost of an increased *S*_*switch*_ threshold.

To separate the effect of using homologous structures from the prediction of effects on interactions, we re-calculated the benchmark statistics using only structures with a very high sequence identity (> = 99%) to the proteins in question. This gave a slightly higher sensitivity (0.42) but with lower accuracy (0.65) and p-value (0.0001) for the desired false positive rate (0.05; See S8 Table), which we believe to be a function of the reduced size of the benchmark.

Finally, to allow for different thresholds for predicting enabling and disabling sites, we split the benchmark in to these two classes and analysed *S*_*switch*_ separately. For our target false positive rate of < = 0.05, enabling and disabling sites gave sensitivities of 0.37 and 0.24 respectively, accuracies of 0.76 and 0.67 respectively, and p-values of << 1 x 10^−6^ and 0.01 respectively (See S9 and S10 Tables). These differences probably reflect the larger number of enabling sites in the benchmark.

Here, for simplicity and the reasons given above, we used the simple *S*_*switch*_ score with a threshold calculated from our combined benchmark. We did not use the optimised classifier, the kinase-deficient or homologue deficient benchmarks, or the separate disabling and enabling benchmarks. Hereafter, we only consider enabling or disabling sites above/below this threshold unless otherwise mentioned. The majority of significant sites have comparatively high sequence identities as might be expected by the nature of the score (>70% have >90% sequence identity, S2 Table).

### Comparison to ΔΔG calculations

There are other methods to calculate or predict the effect of mutations or modifications on protein interactions. Most of these use protein structures of interacting proteins to compute ΔΔG values (i.e. the change of the interaction Gibbs free energy comparing wild-type and modified interactions). We compared our *S*_*switch*_ score to ΔΔGs calculated by FoldX [35] on models we built with Modeller [36] using default parameters. These ΔΔGs were a poor predictor of effects on interactions (True positive rate = 0.01 for a false positive rate 0.05; S6 Table, S4 Fig), highlighting the probable need for manual intervention to get the best results from modelling and energy calculations. For example, the Dynein Ser-88 phosphosite that we predict and is also known to disable homodimerisation (see below) is predicted by FoldX to have a negative ΔΔG (i.e. a more favorable interaction). Inspection shows that the FoldX optimized structure has the two phosphate groups pointing away from each other and accommodated in the dimeric structure instead of pointing towards each other which would prevent dimerisation (S5 Fig). It is possible that more careful consideration of each interface would give better results using FoldX, though this is not practical for the many thousands of Phosphosites considered here.

### Hundreds of new potential phosphosite switches

**Fig 2:**
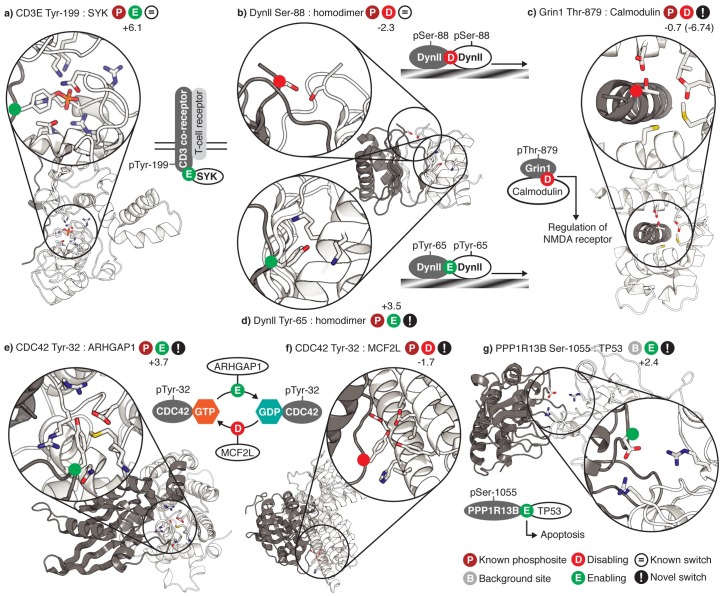
Gallery of phosphosites known or predicted to enable or disable protein interactions. Phosphorylated proteins are shown in grey and the proteins with which they interact are shown in white. The sidechains of phosphosites and the residues with which they interact are shown as sticks coloured by atom type. Phosphosites are indicated by coloured dots on C-alpha atoms. *S*_*switch*_ scores are shown besides enabling or disabling symbols. ***a)*** Phosphorylation of Tyr-199 of an immunoreceptor Tyrosine-based activation motif of the CD3 T-cell co-receptor enables the interaction with Syk Tyrosine by interacting with several positively charged residues [37]. ***b)*** Homo-dimerisation of Dynein light chain is mediated by the phosphorylation of two copies of Ser-88 that are in contact in the homodimer interface. Phosphorylation of both copies of Ser-88 would lead to the repulsion of two negatively charged phosphate groups and is known to lead to the formation of inactive monomers (unable to move along microtubules) in preference to active dimers [40]**. *c)*** Phosphorylation of Thr-879 in Glutamate receptor subunit zeta-1 (GRIN1) is predicted to disable interaction with Calmodulin, and therefore affect regulation of the NMDA receptor [42], since it lies in a highly negatively charged interface (PDB 3bya, to be published). This has a high negative IE (-6.74) but is poorly conserved (f_Cons_ = 0.1) resulting in an *S*_*switch*_ of -0.7, below the threshold. ***d)*** The Tyr-65 phosphosite of human Dynein light chain strongly enables the formation of the Dynein homodimer by interacting with at least two positively charged lysine residues on the opposing face. This enabling effect is doubled because the homodimeric interface is symmetrical [39]. ***e)*** Phosphorylation of Tyr-32 of human CDC42, a small GTPase, enables an interaction with GTPase activator ARHGAP1 through contacts with predominantly positively charged residues [43] and ***f)*** disables an interaction with guanine nucleotide exchange factor MCF2L, since MCF2L residues in contact with Tyr-32 carry a net negative charge, shifting the CDC42 towards its inactive GDP-bound form. The CDC42-MCF2L interaction was modelled on a crystal structure of a human CDC42 homologue interacting with mouse guanine nucleotide exchange factor DBS [44]. ***g)*** The crystal structure of Apoptosis-stimulating of p53 protein 2 (53BP2) interacting with TP53 [45] predicts that phosphorylation of Ser-1055 in the Apoptosis-stimulating of p53 protein 1 (PPP1R13B), which is Aspartate in 53BP2, will enable its interaction with TP53 by interacting with Arg-273 and Arg-248, two arginines which are highly mutated in many human cancers [45].

Considering the 5690 phosphosites at protein-protein interfaces (S2 Table), *S*_*switch*_ predicts 827 (15%) to be enabling and 255 (4%) to be disabling, fractions significantly higher than background (P << 0.01, Fig 1B). Among these are several known enabling switches, such as the Syk Tyrosine kinase SH2 domain bound to an immunoreceptor activation motif [37] (Fig 2A) and Serotonin N-acetyltransferase bound to 14-3-3 zeta [38]. There are also known disabling sites, such as Dynein light chain Ser-88, which is adjacent to a Glutamate and a copy of itself at the dimer interface [39] (Fig 2B) and where phosphorylation leads to inactive monomers [40]. Ser-429 in Mdm2 is also correctly predicted to disable oligomer formation [41]. Of the 123 sites matched to phosphorylated residues visible in at least one 3D interaction interface, 72 are enabling and only two are disabling (the rest are neutral).

Most predicted switches are unknown, including the weakly disabling PKC phosphosite in Glutamate receptor subunit zeta-1, which lies in a negatively charged interface with its regulator Calmodulin (Fig 2C). This has a high negative IE (-6.74) but is poorly conserved (f_Cons_ = 0.1) resulting in an *S*_*switch*_ of -0.7, below the threshold. Examination of the eggNOG group from which f_Cons_ was calculated shows that the majority of the 315 sequences to which this protein was aligned do not align at this point, giving a low f_Cons_. Of those that do, 44% have Threonine at this position.

Novel enabling sites are possibly more difficult to identify since phosphorylation might be required to determine a structure. However, many interactions of known structure are low affinity (possibly half are > 1μM; one third are > 50μM [46]) and high protein concentrations used in structure determination can produce structures without all features necessary for biological interactions. Analysis of our dataset supports this: of the 522 non-redundant phosphosites (in all species) at interfaces that are seen to be phosphorylated in a 3D structure, 16 are unphosphorylated in at least one homologous interface (S3 Table). Thus there are also interesting candidate enabling switches, such as Tyr-65 in human Dynein light chain, predicted to strongly enable homodimer formation by interacting with lysine residues at the interface [39] (Fig 2D). These predicted switches could also be more subtle changes to affinity than (e.g.) SH2 or 14-3-3 domain binding sites, perhaps enhancing or diminishing an interaction that would occur anyway.

Of the 5690 non-redundant sites at protein-protein interfaces, 3225 (57%) represent individual sites that are involved in interactions with multiple partner proteins and 55 represent individual sites that are enabling for one interaction and disabling for another (with another six non-redundant sites being enabling in one protein and disabling in another), suggesting that phosphorylation selects interaction partners. For example, phosphorylation of Tyr-32 of the GTPase CDC42 appears to enable the ARHGAP1 interaction and disable that with the GEF MCF2L (Fig 2E & 2F). Mutation of Tyr-32 in CDC42 is known to abolish exchange activity with GEFs [47], though it is unclear how phosphorylation is involved in this process.

As the set of known phosphosites is incomplete [20], it is likely that many of the background sites are phosphorylated under conditions not yet tested. We thus searched for additional potential switches among these 1.6 million sites. Of these, 31,815 are at a protein-protein interface, of which just 2730 (9%) would, if phosphorylated, be enabling, 780 (2%) would be disabling and 78 (0.2%) would enable some interactions and disable others in the same species. Among these is Ser-1055 in the Apoptosis-stimulating of p53 protein 1, which lies in a long loop directly at the interface with TP53 and interacts with Arg-273 and Arg-248 (Fig 2G). which are mutated in many human cancers [45]. This Serine, which is Aspartate in the closely related TP53BP2, lies in a stretch of three to four Glutamate or Asparate residues in both proteins and is predicted to be a possible Casein kinase phosphorylation site [48,49].

### Validation of potential phosphoswitches

We tested twenty sites with a range of *S*_*switch*_ scores, including known or predicted switching by 13 phosphosites and seven background sites using the yeast two-hybrid system. Based on the few known disabling examples (e.g. Dynein Ser-88 above), we selected five sites (regardless of switch score) for which phosphorylated residues were close to copies of themselves at a homodimer interface. Interestingly, the residue-residue parameters disfavour interactions between unphosphorylated residues (particularly Serine & Threonine) almost as much as between phosphorylated equivalents (Fig 1C), suggesting that their adjacency alone would be insufficient to disable an interface (and indeed at least one of these instances is weakly enabling, see SAT1 below).

We compared the interactions of the natural sequence to those with mutations of the site to Glutamate (commonly used as a phosphosite mimic) or Alanine using the two-hybrid system. Nine of 20 interactions considered gave positive results when using the wild-type clones, a proportion that broadly agrees with the expected sensitivity of the two-hybrid system [50]. Of the sites tested by mutagenesis, four showed definite switching behaviour and five did not (S4 Table). Perhaps highlighting the difficulties in predicting/identifying enabling switches (see above), four of five instances where growth was seen (suggesting an interaction), but no difference could be perceived between wild type and phosphomimic, were predicted enablers (though this finding is not significant; p<0.3 by a hypergeometric distribution). Additionally, while the pair-potential for Glutamate-Glutamate interactions (i.e. our phosphomimetic) is similar to that for pairs of phosphorylated residues except phosphotyrosine (S5 Table), it is also known that Glutamate is an imperfect mimic, particularly for tyrosine-phosphate [51], but also for Serine or Threonine. Indeed, switching behavior for Thr-31 in AANAT/YWAZ (S5 Table) is known to be more apparent when using a chemical phosphomimetic instead of Glutamate [52].

**Fig 3:**
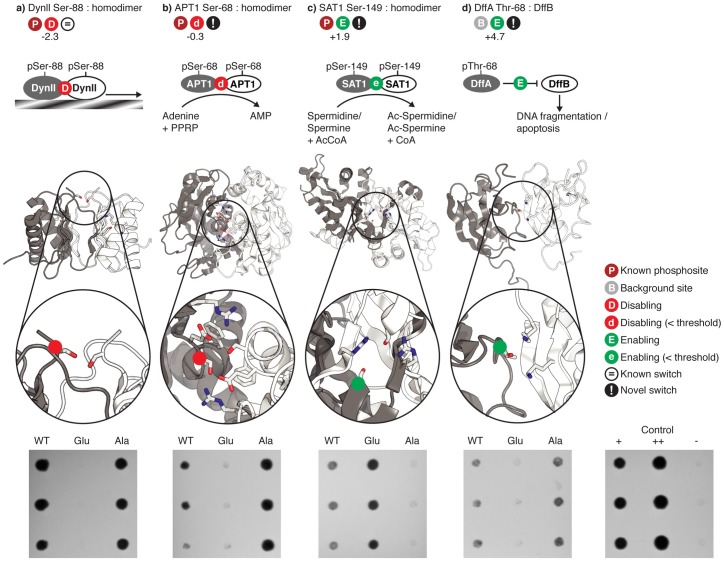
Experimental verification of known and predicted phosphoswitches using wild-type (wt), Glutamate and Alanine mutants in the yeast two-hybrid system. Phosphorylated proteins are shown in grey and the proteins with which they interact are shown in white. The sidechains of phosphosites and the residues with which they interact are shown as sticks coloured by atom type. Phosphosites are indicated by coloured dots on C-alpha atoms. *S*_*switch*_ scores are shown besides enabling or disabling symbols. *a)* the known disabling site Ser-88 in human Dynein [40] is observed to be disabling, with wild-type and Alanine mutants growing but not the Glutamate mutant. *b)* Phosphorylation of Ser-68 in yeast Adenine phosphoribosyltransferase (APT1) was predicted to weakly disable the homodimer interaction by contacting itself and other charged residues in the homodimeric interface [55], which is required for conversion of Adenine to AMP, and a disabling effect was observed. *c)* Phosphorylation of Ser-149 in human diamin acetyltransferase 1 (SAT1) was predicted to enable formation of homodimers [56] required for spermidine/spermine acetylation and was observed to be enabling (wild-type shows growth, Glutamate mutants show stronger growth, Alanine mutant shows no growth). d) Mouse DNA fragmentation factor A (DffA) binds to DffB, inhibiting DNA fragmentation. Phosphorylation of Thr-68 of DffA is predicted to enable interactions with DffB through contacts with two Lysines [57] but is observed to be disabling (wild-type and Alanine mutants show growth, Glutamate does not).

For the known disabling Ser-88 in Dynein (above) both the wild-type and alanine mutants are able to interact, with the Glutamate mutant abolishing the interaction as known (Fig 3A). High-throughput studies in human [53] and yeast [54] identify Ser-68 in yeast Adenine phosphoribosyltransferase from the purine nucleotide salvage pathway to be phosphorylated, and the assay confirms our prediction of a weak disabler (Fig 3B). Another high-throughput site Ser-149 in human diamin acetyltransferase 1 (SAT1) is also enabling as predicted (Fig 3C), with the phosphomimic showing a stronger interaction than wild-type. We also predicted that phosphorylation of Thr-68 of DNA fragmentation factor A (DffA) would enable interactions with DffB. This site is not known to be phosphorylated (i.e. it is a background site), though other sites in the same protein have been identified, including Tyr-75 [34] from the same interface loop. The site does appear to modulate the interface, but is surprisingly disabling (Fig 3D). Inspection shows that the two lysines giving rise to the enabling score are oriented in a way that might preclude effective interactions with the phosphate group and that moreover might lead to steric clashes.

## Discussion

This study is the first large-scale investigation of phosphosites within interacting 3D structures, and has identified hundreds of potential interaction switches. These provide an immediate starting point for additional studies into proteins, interactions and processes affected by such modifications. The phosphoproteome has been estimated to be no more than 22% complete [58]. By this estimate there could be in excess of 4000 enabling or disabling switches across the species we investigated. New candidate switches will be a boost for efforts to unravel the complexity of PTM codes that are critical for fine tuning cellular processes [20]. The fact that so many phosphosites come from high-throughput studies makes structural/mechanistic tools like that presented here important to rank, filter and interpret these data as suggested previously [59]. As with many new technologies in the life sciences, interpretation increasingly lags behind data generation.

Our method to predict the direction of the effect of phosphorylation on a protein-protein interface correctly identified several real enabling or disabling sites, though in some instances we saw no effect or switching in the direction opposite to our predictions. The simple metric does not yet consider the complexities of protein structures, such as conformational rearrangements and steric clashes, multi-faceted interfaces and complex regulation, nor coupling with other modified sites, which determine how phosphorylation might ultimately affect an interaction. It would also benefit from a larger benchmark set of phosphosites known to affect protein-protein interactions, phosphosites known *not* to affect protein-protein interactions, and phosphosites seen directly in protein 3D structures with which we can parameterise our pair-potential scores.

The occurrence of many potential switches in ordered protein regions is surprising given the widely held view that phosphoregulation, particularly in eukaryotes, is predominantly a disordered phenomenon. Indeed, the observation of so many phosphorylation sites at the junction between globular proteins in Eukaryotes (this study) and Prokaryotes [60] and the apparent lack of phosphopeptide binding domains in the latter, suggests that regulation of globular interfaces could be an ancient role for Serine/Threonine kinases, which later diversified into the complex mechanisms—involving disorder and recognition modules—seen in Eukaryotes today.

## Materials and methods

### Phosphoproteome

We took phosphoproteins in five eukaryotes (*H*. *sapiens*, *M*. *musculus*, *D*. *melanogaster*, *C*. *elegans*, *S*. *cerevisae*) from a previous study [24] and identified 258,552 phosphosites in PhosphoSitePlus [61], UniProt [62] (those with experimental evidence only), dbPTM [63] and phospho.ELM [64]. We also extracted phosphorylated Serine, Threonine and Tyrosine residues within known 3D structures [65] which we mapped to UniProt sequences through MUSCLE [66] sequence alignments of SIFTS [67] pairs of PDB and UniProt sequences. For each phosphosite we defined high throughput sites as those seen only in publications reporting 100 or more phosphoproteins. We defined background sites as all 2,068,843 unphosphorylated Serines, Threonines and Tyrosines in the same set of proteins.

To avoid over-counting because of redundancy from sites with equivalents in closely homologous proteins, we grouped all sites (both phosphosites and background) according to their positions in alignments of UniProt UniRef50 sequence groups [68]. We considered potential background sites that were aligned to real phosphosites to be ambiguous and ignored them in our counts and predictions. To avoid grouping poorly aligned sites, we did not group aligned sequences where the number of gaps divided by the sequence length was > = 0.09 (a value deduced by inspection of several hundred phosphoprotein alignments). This gave 223,971 and 1,611,565 non-redundant phosphosites and background sites respectively.

### Phosphosites in 3D structures

We mapped the sequences and sites described above to 3D structures, including interactions with proteins and small-molecules, using Mechismo [32] which uses a non-redundant set of 3D structures of interactions in PDB biological assemblies [69], considers structures of homologues as well as the actual protein in question and transfers positional information via sequence alignments. We used the ‘low’ stringency setting, which identifies the best possible protein-interface for any pair of proteins that interact physically or for which an interaction is known for closely homologous proteins. This setting includes any possible interface of known structure as identified by sequence comparison. In practice, few low identity interfaces are used as the *S*_*switch*_ score (below) down-weights switches arising from more remote homologues. As in Mechismo itself, we do not construct protein models, but transfer residue contacts from the template structure to a target sequence (even if matched amino acids are different). In cases where multiple templates were available for a site at a particular interface (as a result of different alignments between UniProt and the PDB, which can come from SIFTS or from BLASTP within Mechismo), we took the most significant score (either enabling or disabling).

3D interaction structures with phosphorylated Serine, Threonine or Tyrosine (PDB SEP, TPO and PTR) residues seen directly in interfaces, from any species, were compared to similar interfaces (at least 50% sequence identity across at least 50% of the sequence, and at least 50% interface residues in common after alignment) to identify homologous interactions with unphosphorylated residues at the equivalent position. Multiple phosphorylated residues at the same position in the same interface group were counted only once.

### Disorder and exposure

We defined intrinsically disordered residues as those where the mean IUPred long disorder [70] of the matching fragment residue over a sliding window of eleven residues was ≥ 0.5. We defined residues as buried when the side-chain accessible surface area of the aligned residue in the structural template was < 5Å^2^ and exposed otherwise (using NACCESS [71]).

### Switch score

We defined the switch score as:

$$S_{switch}=\mathrm{IE}\;x\;f_{\mathrm{ID}}\;x\;f_{\mathrm{Cons}}$$

Where IE (Interaction Effect) is the sum of changes in residue pair-potentials upon phosphorylation [32] (S5 Table), f_ID_ is the minimum of the fraction of identical residues in the alignment of either sequence with its structural template, and f_Cons_ is the fraction of sequences in the alignment of the animal or fungus (i.e. opisthokont) eggNOG 4.5 [72] orthologous group that have a residue of the same amino acid type or Aspartate or Glutamate aligned to the site. For homodimeric interactions, the site was assumed to be phosphorylated in both copies of the protein. For sites for which f_Cons_ was unavailable (i.e. not aligned to any other sequence), we used the average f_Cons_ of all Serines, Threonines and Tyrosines in proteins of the same species.

### Benchmark for protein switches

We defined the positive benchmark set by extracting all 1339 phosphosites from UniProt ‘MOD_RES’ records from the species studied here and where the annotated text gave indications of binding/interaction (“bind*” or “interact*”) and/or mentioned multimerisation or at least one additional protein by gene name. We then inspected these and marked relationships as enabling, disabling, phosphorylation/dephosphorylation or unknown which left 795 phosphosite-interaction pairs in 222 proteins. We also downloaded regulatory sites from PhosphoSitePlus [34] and extracted protein interaction pairs marked as being induced or disrupted by a phosphosite, given 5225 interaction pairs involving 3323 sites in 1588 proteins from 13 species.

We defined the negative benchmark set by shuffling positions in this set, along with their interactors and the given effect, to a random position in the same protein and did this ten times for each site. In doing so we preserved the distribution of surface exposures of these sites as described previously [32]. This gave 41813 site-interaction pairs involving 28441 sites in the same set of proteins. We mapped the benchmark sites and their interactors to interaction structures and discarding unmapped pairs, leaving 122 unique positives and 224 negatives (S1 Table). We then evaluated classifier performance using the R package 'ROCR' [73]. To account for possible bias towards enabling sites from kinase-substrate interactions, we classified all interactors as kinases when they matched to a protein kinase domain in Pfam [74] (specifically, Pfam accession PF00069) and re-calculated the benchmark statistics using this reduced set.

### Logistic regression to optimize performance

To optimise the combination of IE, f_ID_ and f_Cons_, we applied logistic regression to our benchmark using R [75]. We balanced the benchmark data by randomly undersampling the negative set, ran five-fold cross-validation, repeated this 100 times, and took the means of the following summary statistics to evaluate the model: Area Under the Curve (AUC), threshold that gave a False Positive Rate (FPR) of < = 0.05, and the accuracy, True Positive Rate (TPR), True Negative Rate (TNR) and Positive Predictive Value (PPV) at this threshold. We then applied logistic regression to the full benchmark set.

### Comparison with FoldX

For each phosphosite interaction in our benchmark, using the same template structure as for Sswitch, we used Modeller [36] to build a model of the unphosphorylated interaction and FoldX [35] to produce the phoshorylated version. We then used FoldX to calculate the ΔΔG between these two models.

### Significance calculations

We calculated the significances of the differences of distributions (accessible surface area, f_Cons_) of phosphosites and of background sites with Wilcoxon-Mann-Whitney rank sum tests. We used chi-square tests to calculate the significances of the differences in the fractions of phosphosites and of background sites under the various binary classifiers (ordered, mapped to structure, exposed, in an interaction interface, and enabling or disabling). In all cases, P was << 0.01. We calculated p-values for the selected score thresholds on the benchmark using a two-sided Fisher's exact test.

### Open reading frame cloning

A total of 70 open reading frames encoding putative phospo-switchable proteins and their interactors were obtained as sequence optimised synthetic clones flanked by attb-Gateway sites (GeneArt/ Invitrogen). All clones were Gateway-cloned into the Donor vectors pDONR221 or if necessary into pDONR/Zeo by Gateway BP-reaction and subsequently by LR-reaction into the Y2H bait and prey vectors pDEST32 and pDEST22 respectively for the Yeast two Hybrid experiments. All constructs were sequence verified.

### Code

All code is available from the Mechismo website, mechismo.russelllab.org/downloads.

### Yeast two-hybrid assays

We performed two-hybrid assays following an altered “Testing specific Two-Hybrid interaction” protocol of the ProQuest™ Two-Hybrid System Handbook (Invitrogen). Briefly, all interaction pairs (wild-type, Glutamate- and Alanine-mutants) were double-transformed into yeast strain MaV203 (Invitrogen, MaV203 Competent Yeast Cells, Library Scale cat# 11281–011). Colonies from each transformation were grown on 15-cm plates of synthetic complete media lacking leucine and tryptophan (Sc-Leu-Trp). After 2–3 days 3 individual colonies of each transformation were picked and suspended in 100 μl autoclaved saline in a 96-well PCR plate. From here they were replicated by 96-needle replicator onto rectangular SC-Leu-Trp agar plates lacking histidine and containing three different concentrations (10, 25, 50 mM) of 3-aminotriazol (3AT). 2–5 days after plating interaction phenotypes were assessed. For phosphotyrosine sites we also tested the Tyrosine to Alanine-Glutamate mutation which is proposed to be a better mimic of phosphotyrosine [51]. For homodimeric interactions, colonies where both copies of the protein contained the phosphomimetic were examined.

## Supporting information

S1 Fig The distributions of side-chain accessible surface area for phosphosites (red line, left-hand y-axis) and background sites (grey line, right-hand y-axis) are significantly different (P << 0.01), with phosphosites more likely to be exposed. The left and right-hand axes are scaled to the number of non-redundant phosphosites and background sites, respectively, that were mapped to structures.
(TIF)

S2 Fig The distributions of conservation scores of phosphosites (red line, left-hand y-axis) and background sites (grey line, right-hand y-axis) mapped to interfaces are significantly different (P << 0.01), with phosphosites more likely to be conserved. The left and right-hand axes are scaled to the number of non-redundant phosphosites and background sites, respectively, that were mapped to interfaces of interaction structures.
(TIF)

S3 Fig Receiver Operator Characteristic (ROC) curves showing the ability of absolute interaction effect (abs(IE)), similarity of the query to the template structure (f_ID_) and the conservation of the site across orthologues (f_Cons_) to identify phosphosite switches irrespective of the direction of the effect. (TIF)

S4 Fig Top panel: Receiver Operator Characteristic (ROC) curves showing the ability of *S*_*switch*_ to identify enabling or disabling effects of known phosphosite switches, the change in performance by including the interaction effect (IE, = sum of pair-potentials), similarity of the query to the structure template (f_ID_), and the conservation of the site across orthologues (f_Cons_), along with performance of logistic regression (LR final) using the same features and the performance of ΔΔG calculated by FoldX. Bottom panel: Precision-Recall curves for the same set of predictors.
(TIF)

S5 Fig The Dynein Ser-88 phosphosite is known to disable homodimerisation [40] but FoldX gives a negative ΔΔG (-4.83 Kcal/mol), with energy minimisation causing the two Serines that point towards each other in the unphosphorylated structure (top) to point away from each other when phosphorylated (bottom) rather than preventing dimerisation [35]. (TIF)

S1 Table Phosphoswitch benchmark set. (Data file 'S1_table_benchmark.txt'.) Please check the given effect in original sources (UniProt and PhosphoSitePlus) in case of new information and updated annotations. Tab-separated-variables format with the following columns:
set: ‘positive’ or ‘negative’protein: name and UniProt accession of protein containing the sitesite: wild-type amino acid, sequence position, and modificationsite+-7AA: the given site (lowercase) plus flanking sequence (uppercase) from up to seven amino acids before and after.interactor: name and UniProt accession of interacting proteineffect: known effect of phosphorylation of the site on the interactiontemplate: PDB identifier and the two sections which specify the interaction. If the interaction is only present in a biological assembly, the PDB-identifier is suffixed with the assembly and model numbers of the two sections.pdbres: chain identifier, residue sequence number, insertion code and three-letter amino-acid code for the template residue aligned to the site.IEf_ID_f_Cons_*S_switch_*ddGkinase–indicates whether or not the given interactor is a kinase.
(TXT)

S2 Table *S_switch_* scores for phosphosites and background. (Data file 'S2_table_sswitch.txt'.) Tab-separated variables with the following columns:
protein: name and UniProt accession of protein containing the sitespeciessite: wild-type amino acid, sequence position, and modificationsite+-7AA: the given site (lowercase) plus flanking sequence (uppercase) from up to seven amino acids before and after.phosphosite source databasePubMed identifiers of referencesinteractor: name and UniProt accession of interacting proteintemplate: PDB identifier and the two sections which specify the interaction. If the interaction is only present in a biological assembly, the PDB-identifier is suffixed with the assembly and model numbers of the two sections.pdbres: chain identifier, residue sequence number, insertion code and three-letter amino-acid code for the template residue aligned to the site.IEf_ID_f_Cons_*S_switch_*uniref50apos: name and UniProt accession of reference sequence of UniRef50 group to which the protein belongs, along with the position of the site in the sequence alignment of that groupthroughput: ‘best’ throughput of any reference that defines this site, ‘stp’ = single throughput (one phosphoprotein identified in the publication), ‘ltp’ = low throughput (2–19 phosphoproteins), ‘mtp’ = medium throughput (20–99 phosphoproteins), ‘htp’ = high throughput (> = 100 phosphoproteins), ‘utp’ = unknown throughput (publication could not be traced), ‘N/A’ = not applicable.
(TXT)

S3 Table Phosphorylated residues seen directly in interfaces of 3D structures and their unphosphorylated equivalents in homologues of known structure. (Data file 'S3_table_pdbphos.txt'.) Tab-separated variables with the following columns:
id_res: residue unique identifierid_res_nr: residue non-redundant identifierstruct1: PDB identifier and the two sections which specify the interaction. If the interaction is only present in a biological assembly, the PDB-identifier is suffixed with the assembly and model numbers of the two sections.pdbres1: chain identifier, residue sequence number, insertion code and three-letter amino-acid code for the template residue aligned to the site.aa1: three-letter amino acid codephosStruct: structure information of the best (highest percent sequence identity) homologue of known structure that also has a phosphorylated residue at the same positionphosPdbresphosAAphosPcid: percent sequence identity between phosStruct and struct1unphosStruct: structure information of the best (highest percent sequence identity) homologue of known structure that has an unphosphorylated residue at the same positionunphosPdbresunphosAAunphosPcid: percent sequence identity between unphosStruct and struct1.
(TXT)

S4 Table Summary of results of experimental validation. Predicted effects are given in brackets when *S*_*switch*_ is below the threshold. ‘wt’, ‘E’, ‘A’, summarise the growth seen for the wild-type, Glutamate and Alanine mutants respectively. For homodimer interactions, ‘E/A’ represents Glutamate in the bait and Alanine in the prey, and vice-versa for ‘A/E’. ‘+’ = growth with respect to negative control, ‘-‘ = no growth. Superscript ‘*’ in the ‘site’ column denotes known switches, ‘^’ denotes sites chosen because they point at a second copy of themselves across a homodimeric interface and ‘b’ denotes background sites.
(PDF)

S5 Table Matrix of pair-potential scores that compare the frequency of pairs of contacting residues at interfaces to a random model. (Data file 'S5_table_pair_potentials.txt'.)
(TXT)

S6 Table Statistics for the predictors run on the entire benchmark set. Tab-separated variables with the following columns:
name: name of the predictor, where: 
'LR [number]' = an individual fold of cross-validation'LR mean' = the means of the statistics from cross-validation'LR sd' = the standard deviations of the statistics from cross-validation'LR final' = logistic regression using the entire benchmark set for trainingIE = prediction using Interaction Effect onlyIE x fID = prediction using Interaction Effect multiplied by the fraction of identical residues between the query proteins and the structure templateIE x fID x fCons = prediction using Interaction Effect multiplied by the fraction of identical residues between the query proteins and the structure template multiplied by residue conservationddG–prediction using ΔΔG calculated with FoldXIntercept: intercept coefficient from logistic regression analysisRocIE: coefficient for IE from logistic regression analysisfID: coefficient for fID from logistic regression analysisfCons: coefficient for fCons from logistic regression analysiscut: the score threshold above which the false positive rate is < = 0.05fpr: the false positive rate at the given thresholdtpr: the true positive rate at the given thresholdtnr: the true negative rate at the given thresholdacc: the accuracy at the given thresholdppv: the positive predictive value at the given thresholdp: the p-value from the two-sided Fisher's exact test at the given threshold.
(TXT)

S7 Table Statistics for the predictors run on the benchmark set with kinase interactors removed. Columns as per S6 Table.
(TXT)

S8 Table Statistics for the predictors run on the benchmark set with fID < 0.99 removed. Columns as per S6 Table.
(TXT)

S9 Table Statistics for the predictors run on the benchmark set including only enabling switches. Columns as per S6Table.
(TXT)

S10 Table Statistics for the predictors run on the benchmark set including only disabling switches. Columns as per S6 Table.
(TXT)
