# Supplementary figures and images for: Systematic identification of phosphorylation-mediated protein interaction switches
Source: PLoS Comput Biol. 2017 Mar 27;13(3):e1005462. doi: 10.1371/journal.pcbi.1005462 (PMC5386296; doi:10.1371/journal.pcbi.1005462)

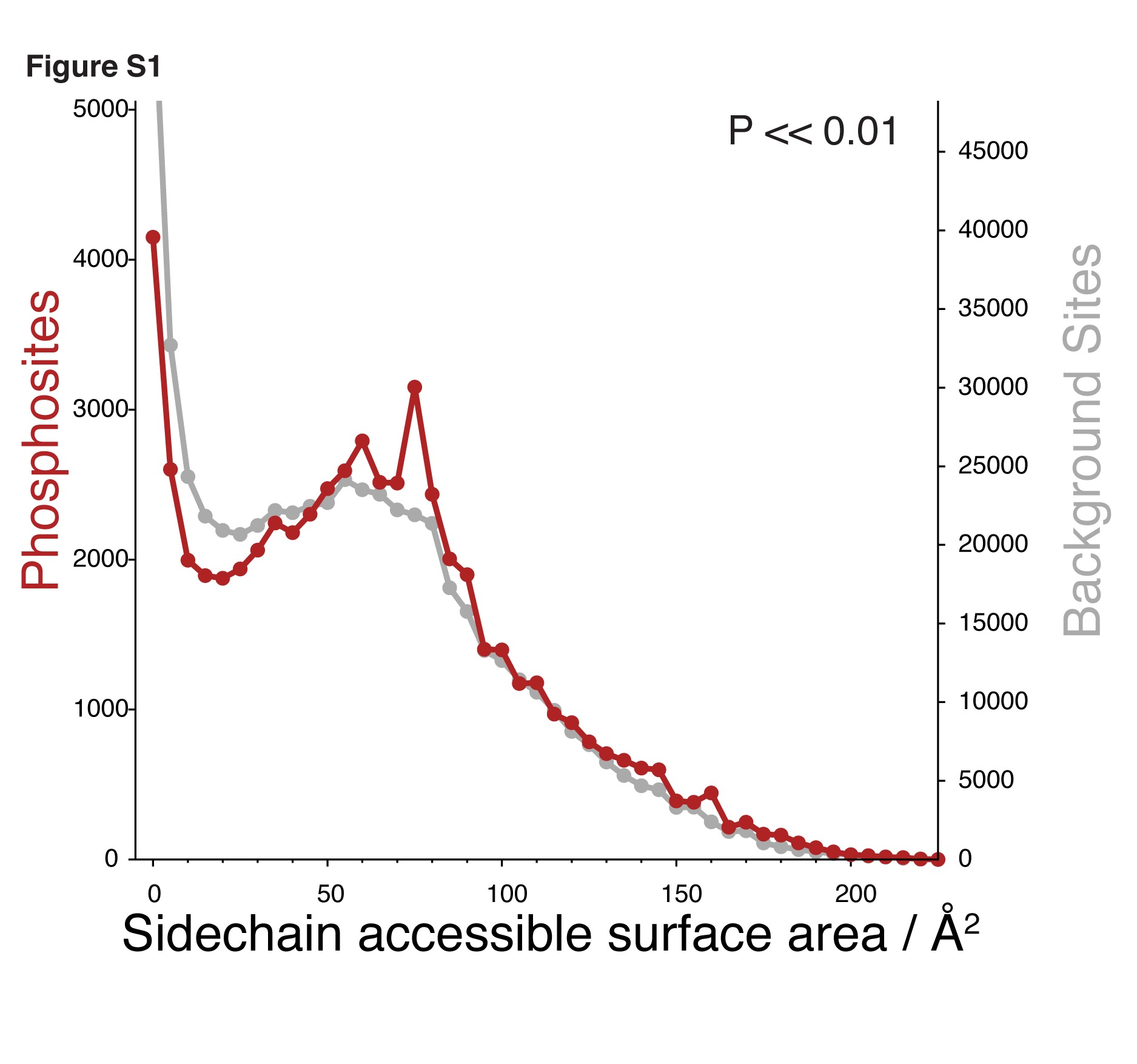

Supplement: S1 Fig — The left and right-hand axes are scaled to the number of non-redundant phosphosites and background sites, respectively, that were mapped to structures. (TIF) [file pcbi.1005462.s001.tif]

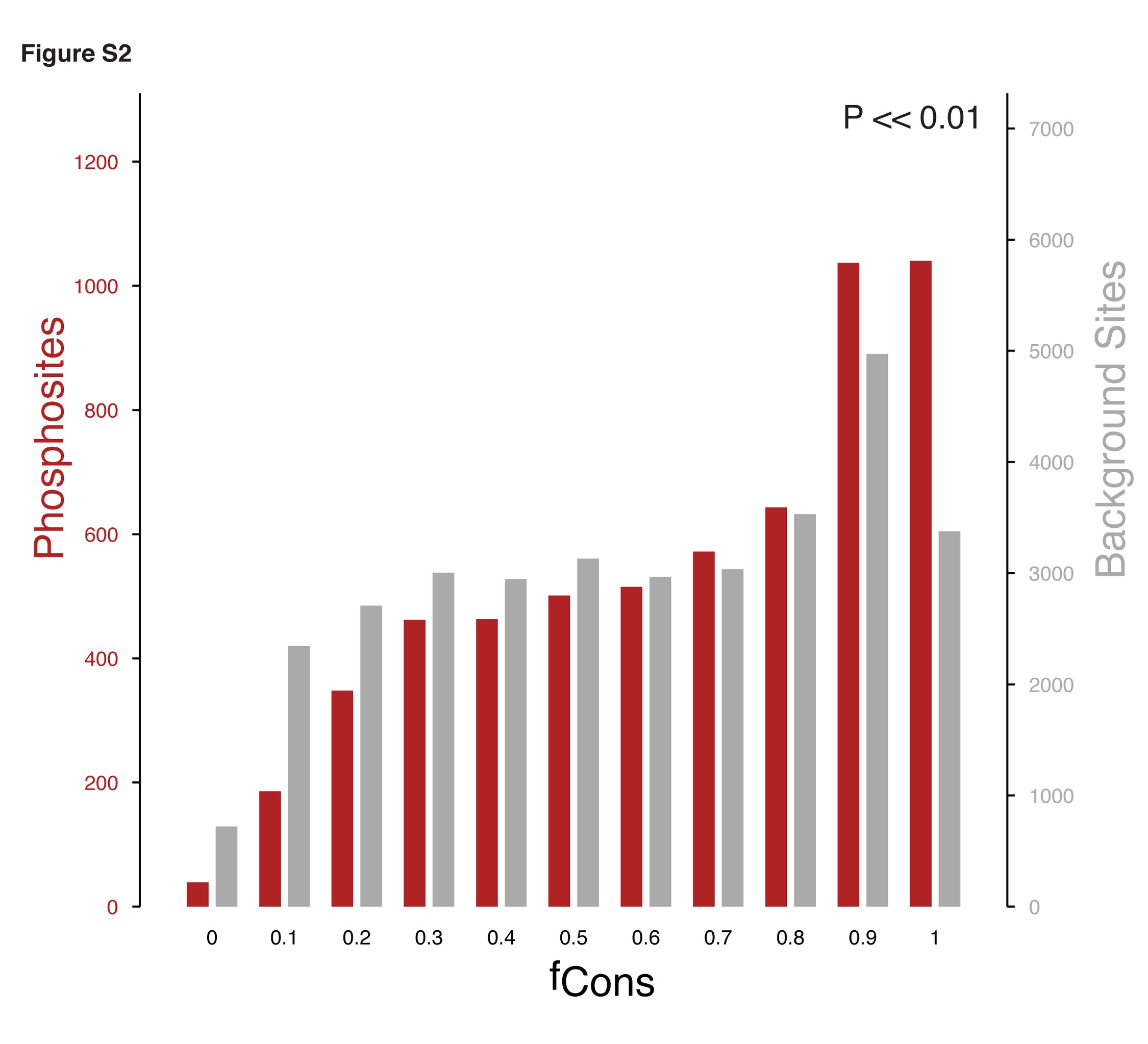

Supplement: S2 Fig — The left and right-hand axes are scaled to the number of non-redundant phosphosites and background sites, respectively, that were mapped to interfaces of interaction structures. (TIF) [file pcbi.1005462.s002.tif]

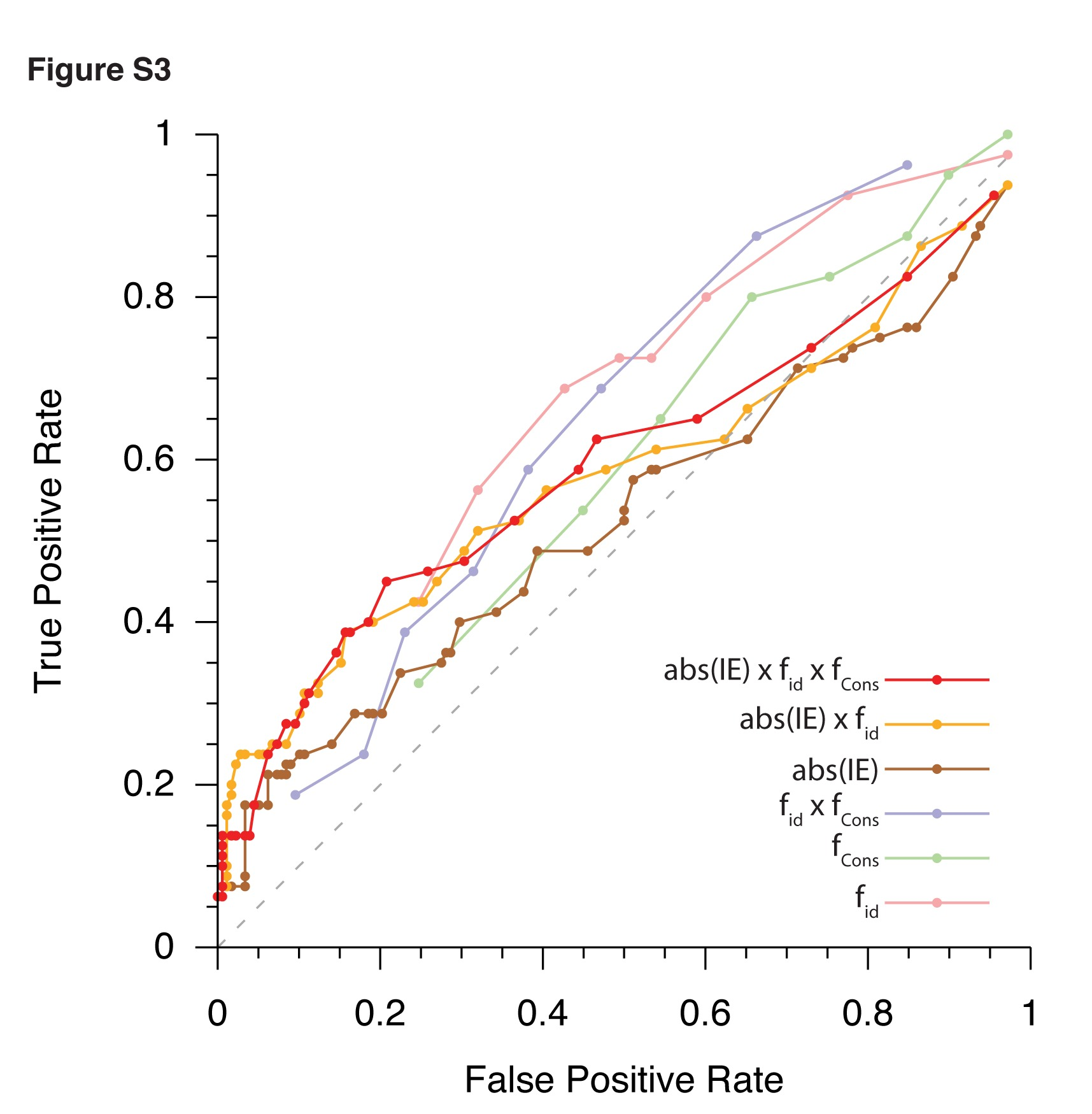

Supplement: S3 Fig — (TIF) [file pcbi.1005462.s003.tif]

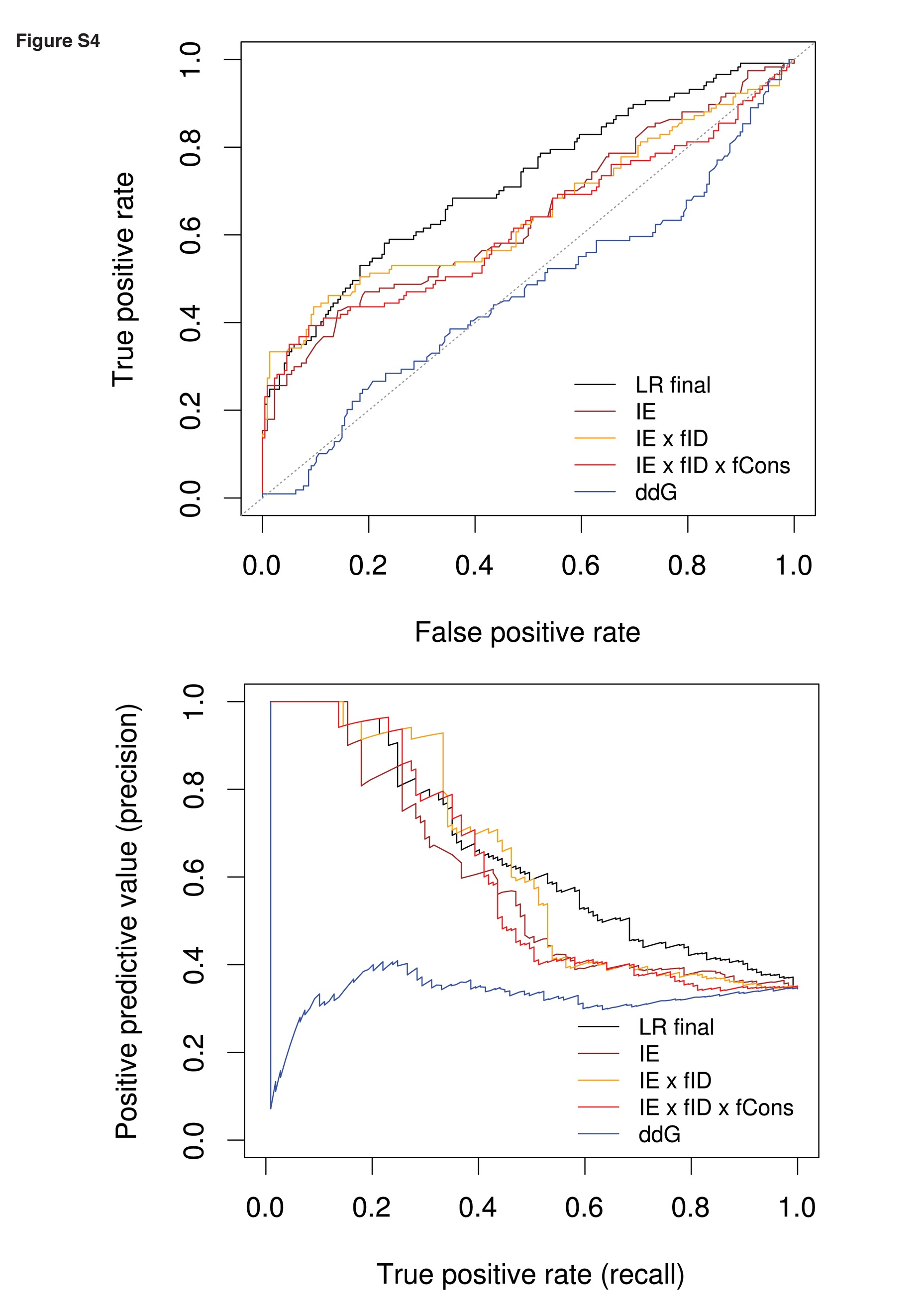

Supplement: S4 Fig — Bottom panel: Precision-Recall curves for the same set of predictors. (TIF) [file pcbi.1005462.s004.tif]

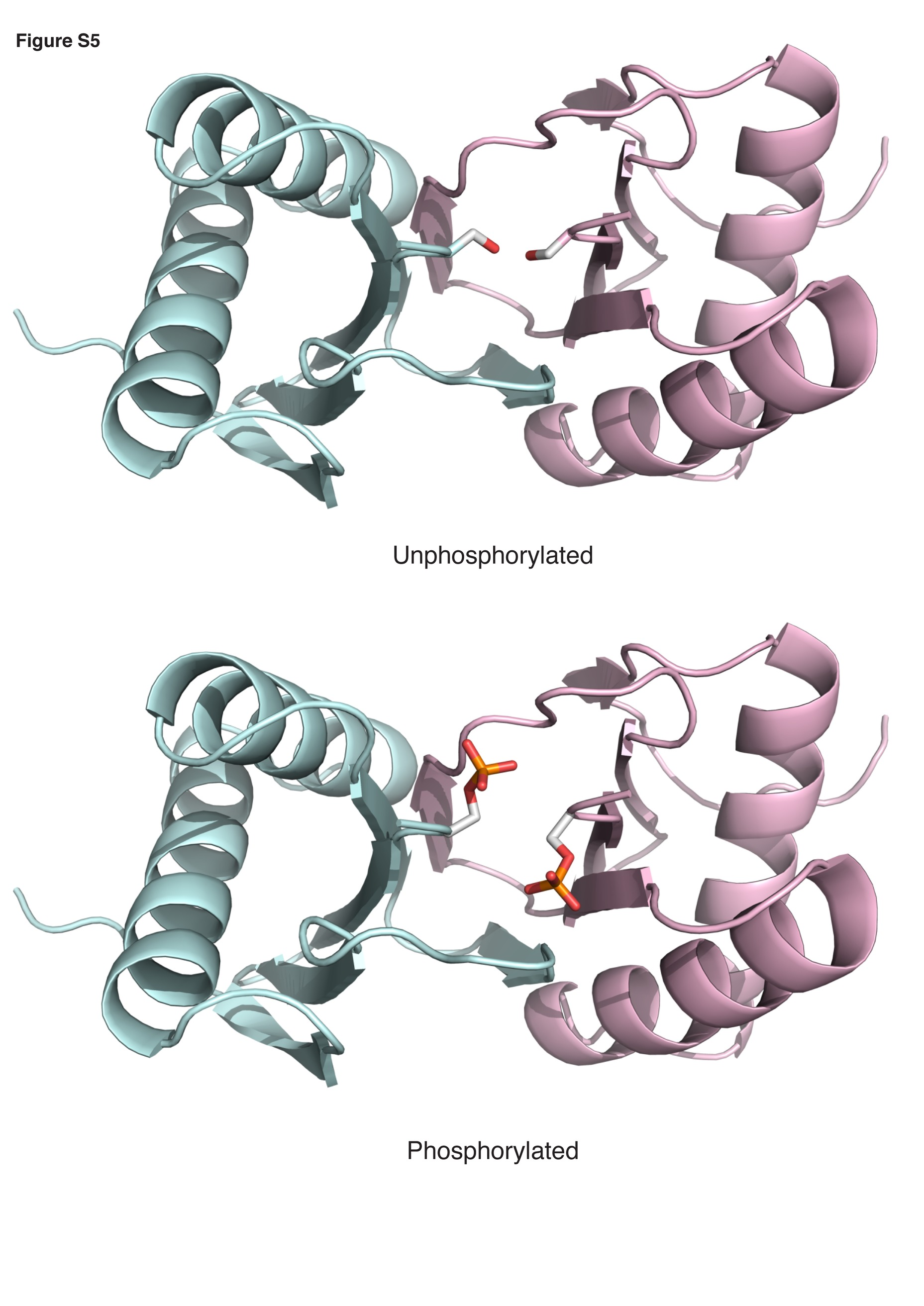

Supplement: S5 Fig — (TIF) [file pcbi.1005462.s005.tif]
