# Supplementary material for: Systematic identification of phosphorylation-mediated protein interaction switches
Source: PLoS Comput Biol. 2017 Mar 27;13(3):e1005462. doi: 10.1371/journal.pcbi.1005462 (PMC5386296; doi:10.1371/journal.pcbi.1005462)
Supplement: S4 Table — Predicted effects are given in brackets when Sswitch is below the threshold. ‘wt’, ‘E’, ‘A’, summarise the growth seen for the wild-type, Glutamate and Alanine mutants respectively. For homodimer interactions, ‘E/A’ represents Glutamate in the bait and Alanine in the prey, and vice-versa for ‘A/E’. ‘+’ = growth with respect to negative control, ‘-‘ = no growth. Superscript ‘*’ in the ‘site’ column denotes known switches, ‘^’ denotes sites chosen because they point at a second copy of themselves across a homodimeric interface and ‘b’ denotes background sites. (PDF) [file pcbi.1005462.s009.pdf]

| Protein | Site | Interactor | Predicted | IE | $f_{ID}$ | $f_{Cons}$ | $S_{switch}$ | wt | E | A | E/A | A/E | Observed |
|---|---|---|---|---|---|---|---|---|---|---|---|---|---|
| DYNLL, P63167 | S88*^ | Self | **Disabling** | -3.14 | 1.00 | 0.73 | -2.3 | + | - | + | - | - | **Disabling** |
| AANAT, Q16613 | T31* | YWHAZ, P63104 | **Enabling** | 4.59 | 0.80 | 0.83 | 3.0 | - | - | - | | | No growth |
| UPF3B, Q9BZI7 | Y98 | UPF2, Q9HAU5/768-1015 | **(Disabling)** | -0.11 | 1.00 | 0.91 | -0.1 | | | | | | Autoactivation |
| GSP1, P32835 | S181 | YRB1, P41920/71-201 | **(Enabling)** | 1.03 | 0.95 | 0.14 | 0.1 | | | | | | Autoactivation |
| SSA3, P09435/4-545 | T417 | SSE1, P32589 | **Enabling** | 5.68 | 0.80 | 0.67 | 3.0 | + | + | + | | | Not switched |
| GSK3B, P49841/56-340 | S219 | Self | **Enabling** | 2.83 | 1.00 | 1.00 | 2.8 | + | + | + | + | + | Not switched |
| ENO1, P00924 | S404 | Self | **Disabling** | -2.13 | 1.00 | 0.92 | -2.0 | + | + | + | + | + | Not switched |
| SNF1, P06782/46-319 | S211 | Self | **(Disabling)** | -1.11 | 0.93 | 0.30 | -0.3 | - | - | - | - | - | No growth |
| APRT, P07741 | S66^ | Self | **Enabling** | 3.60 | 1.00 | 0.39 | 1.4 | + | + | + | - | + | Not switched |
| APT1, P49435 | S68^ | Self | **(Disabling)** | -0.73 | 0.99 | 0.39 | -0.3 | + | - | + | ++ | + | **Disabling** |
| G3BP2, Q9UN86/1-139 | T111^ | Self | **(Disabling)** | -1.39 | 0.76 | 0.93 | -1.0 | - | - | - | - | - | No growth |
| SAT1, P21673 | S149^ | Self | **Enabling** | 2.29 | 1.00 | 0.85 | 1.9 | + | ++ | - | - | - | **Enabling** |
| GOT1, P17174 | Y71 | Self | **Enabling** | 7.26 | 1.00 | 0.95 | 6.9 | + | + | + | + | + | Not switched |
| CDC42, P60953 | T35[b] | MCF2L, O15068/623-964 | **Disabling** | -3.62 | 0.93 | 0.95 | -3.2 | - | - | - | | | No growth |
| GNAI3, P08754/33-348 | T182[b] | RGS8, P57771/46-176 | **Enabling** | 4.66 | 1.00 | 0.92 | 4.3 | - | - | - | | | No growth |
| NUDT21, O43809/21-227 | T166[b] | CPSF6, Q16630/81-173 | **(Disabling)** | -0.38 | 0.98 | 0.92 | -0.3 | - | - | - | | | No growth |
| Dffa, O54786/1-100 | T68[b] | Dffb, O54788/1-87 | **Enabling** | 4.70 | 0.99 | 1.00 | 4.7 | + | - | + | | | **Disabling** |
| PPP1R13B, Q96KQ4/888-1080 | S1055[b] | TP53, P04637/97-287 | **Enabling** | 5.86 | 0.81 | 0.50 | 2.4 | - | - | - | | | No growth |
| STX1A, Q16623/2-248 | S139[b] | STXBP1, P61764 | **Enabling** | 8.04 | 0.93 | 0.37 | 2.8 | | | | | | Autoactivation |
| STXBP1, P61764 | T52[b] | STX1A, Q16623/2-248 | **(Enabling)** | 5.19 | 0.93 | 0.33 | 1.6 | | | | | | Autoactivation |
